# Alemtuzumab and thrombotic thrombocytopenic purpura: Analysis of an international surveillance database and systematic literature review^☆^

**DOI:** 10.1016/j.transci.2025.104081

**Published:** 2025-01-27

**Authors:** Jeremy W. Jacobs, Thomas C. Binns, Danielle Schlafer, Jennifer S. Woo, Garrett S. Booth, Brian D. Adkins

**Affiliations:** a Department of Pathology, Microbiology & Immunology, Vanderbilt University Medical Center, Nashville, TN, USA; b Department of Laboratory Medicine, Yale School of Medicine, New Haven, CT, USA; c Department of Pharmaceutical Services, Emory Healthcare, Atlanta, GA, USA; d Department of Pathology, City of Hope National Medical Center, Irvine, CA, USA; e Department of Pathology, University of Texas Southwestern Medical Center, Dallas, TX, USA

**Keywords:** Thrombotic microangiopathy, Thrombotic thrombocytopenic purpura, Alemtuzumab, Drug reaction, Adverse event, Safety, Monitoring

## Abstract

**Objectives:**

Thrombotic thrombocytopenic purpura (TTP) is a thrombotic microangiopathy associated with severe deficiency in ADAMTS13. ADAMTS13 deficiency may be secondary to absent or dysfunctional protein production due to mutations in the *ADAMTS13* gene (congenital TTP) or autoantibody-mediated clearance and/or inhibition (immune-mediated TTP). This autoimmunity may, albeit rarely, occur secondary to certain medications (eg, ticlopidine). Recent case reports have implicated alemtuzumab (LETRADA), a monoclonal antibody that selectively inhibits CD52, as a cause of secondary TTP. We aimed to characterize all reports of TTP potentially associated with alemtuzumab.

**Methods:**

We performed a cross-sectional analysis of the United States Food and Drug Administration’s Adverse Event Reporting System (FAERS) database as of 21 November 2024 and systematically reviewed the literature as of 03 September 2024 for all reported cases of secondary TTP potentially associated with alemtuzumab. Patient demographics, therapy indications, associated medications, and outcomes were abstracted.

**Results:**

We identified 49 reports of TTP possibly related to alemtuzumab administration since 01 January 2001 in the FAERS database, 9 of which resulted in death. Most patients (n = 31) were receiving alemtuzumab for multiple sclerosis (MS), while 8 reports were in patients undergoing hematopoietic stem cell transplantation. We identified two additional cases in the literature review in patients receiving alemtuzumab for MS.

**Conclusions:**

In conjunction with studies of the United Kingdom’s and European Union’s pharmacovigilance databases, these results support the current package insert for alemtuzumab in which TTP is listed as a “warning and precaution”. Increased awareness of this possible side effect, and prolonged monitoring, is warranted.

## 1. Introduction

Thrombotic microangiopathy (TMA) is characterized by endothelial damage and microthrombi formation in capillaries and arterioles leading to the development of microangiopathic hemolytic anemia (MAHA), thrombocytopenia, and end-organ ischemia [1]. TMA is not one disease; rather, it is the broad term that encompasses disorders with thrombocytopenia and MAHA. These conditions may be idiopathic or secondary to a variety of inciting factors (eg, malignancy, hematopoietic progenitor cell transplantation, medications) [2-4].

Drug-induced TMA (DI-TMA) is the general term that describes cases that were previously categorized as drug-induced thrombotic thrombocytopenic purpura and drug-induced hemolytic uremic syndrome (HUS) [5,6]. The pathophysiology of DI-TMA is multifactorial, and may include the development of autoimmunity, drug-dependent antibodies, and endothelial toxicity [5]. In contrast to thrombotic thrombocytopenic purpura (TTP), which is defined as a severe deficiency of plasma ADAMTS13 (a disintegrin-like metalloproteinase with thrombospondin motif type 1 member 13) activity secondary to absent or dysfunctional protein production due to mutations in the *ADAMTS13* gene (ie, congenital TTP) or autoantibody-mediated clearance and/or inhibition (ie, immune-mediated TTP) [7], most cases of DI-TMA are not associated with severe ADAMTS13 deficiency/inhibition. However, in cases where ADAMTS13 deficiency/inhibition occurs in the setting of a drug, the 2023 American Society for Apheresis (ASFA) guidelines indicate that a diagnosis of drug-induced or secondary TTP is more appropriate than DI-TMA [5]. Ticlopidine is one such example that can present with significantly reduced ADAMTS13 activity and detectable inhibitors. In these cases, the TMA should be managed similarly to TTP [5,8].

Alemtuzumab (LEMTRADA), a humanized monoclonal antibody directed against CD52, is another agent that has recently been implicated as a cause of DI-TMA with an immune-mediated mechanism resulting in significant deficiency of ADAMTS13 (ie, secondary TTP) [9, 10]. Indeed, this has even been referred to as “alemtuzumab-induced immune-mediated thrombotic thrombocytopenic purpura” [9], illustrating the challenge with accurately distinguishing and classifying this phenomenon. Importantly, alemtuzumab has not historically been included in guidelines for monitoring and treating patients for potential DI-TMA or secondary TTP. As such, the potential association between alemtuzumab and DI-TMA or secondary TTP is unknown. However, various studies have demonstrated that alemtuzumab is associated with autoimmunity, including secondary immune-mediated cytopenias [11, 12].

Alemtuzumab is currently marketed in the United States (US) and Europe as LEMTRADA for patients with relapsing-remitting multiple sclerosis (MS) [13,14]. It may also be used for patients with chronic lymphocytic leukemia/small lymphocytic lymphoma where it was historically marketed as Campath and MabCampath, and as a component of conditioning regimens for hematopoietic stem cell transplantation (HSCT) to prevent graft-versus-host disease (GVHD) [15]. The package insert recommends that prescribers monitor patients at periodic intervals for 48 months post-therapy for autoimmunity [13], and includes TTP as a “warning and precaution”. Alemtuzumab also carries a “boxed warning” for autoimmune conditions [13]; these are hypothesized to arise following the binding of alemtuzumab to CD52 (a surface protein found on T and B lymphocytes, natural killer cells, monocytes, and macrophages) [13,16], resulting in the depletion of T and B cells via antibody-dependent cytolysis and subsequent immunologic modulation and reconstitution [13,16-19].

Despite these warnings, contemporary awareness of this potential association remains limited; as such, we aimed to characterize all reports of possible alemtuzumab and TTP to ascertain if recent case reports may reflect a more prevalent concern. This may have particular relevance for both prescribers of alemtuzumab and those who may be consulted for the management of patients with DI-TMA or TTP (eg, apheresis providers).

## 2. Materials and methods

The objective of this study was to assess all reports in which alemtuzumab may have been associated with TTP. To address this, we analyzed the United States (US) Food and Drug Administration (FDA) Adverse Event Reporting System (FAERS) database [20] and performed a systematic literature review. This cross-sectional analysis did not require ethics board review as all data were publicly available.

The FAERS database serves as an international reporting system comprised of adverse event and medication error reports submitted to the US FDA [21]. This database is utilized for post-marketing safety surveillance for drugs and therapeutic biologics [21]. The FAERS database was queried on 21 November 2024 for reports of TTP with alemtuzumab since January 1, 2001. As of the search date, data were updated through 30 September 2024 in the FAERS database, with 29,661,136 total reports, of which 16,394,188 were deemed serious (defined by FAERS as an outcome that includes death, hospitalization, life-threatening, disability, and/or congenital anomaly), and 2,683,911 resulted in death. We used all generic (alemtuzumab) and any current or previously associated trade names (Campath, MabCampath, Lemtrada) for alemtuzumab available in the FAERS database. We used the FAERS code “Thrombotic Thrombocytopenic Purpura” to identify all cases of TTP. We reviewed any corresponding references for case report details and included these in the final analysis regardless of clinical scenario. Data abstracted included patient demographics, therapy indications, associated medications, and outcomes, where available. Because TMA due to transplantation or calcineurin inhibitor (CI) use (transplant-associated or CI-induced TMA respectively) could be confounded with a diagnosis of TTP, we also performed a subset analysis of only the FAERS TTP cases that did not report transplantation or CI use.

**Fig. 1.**
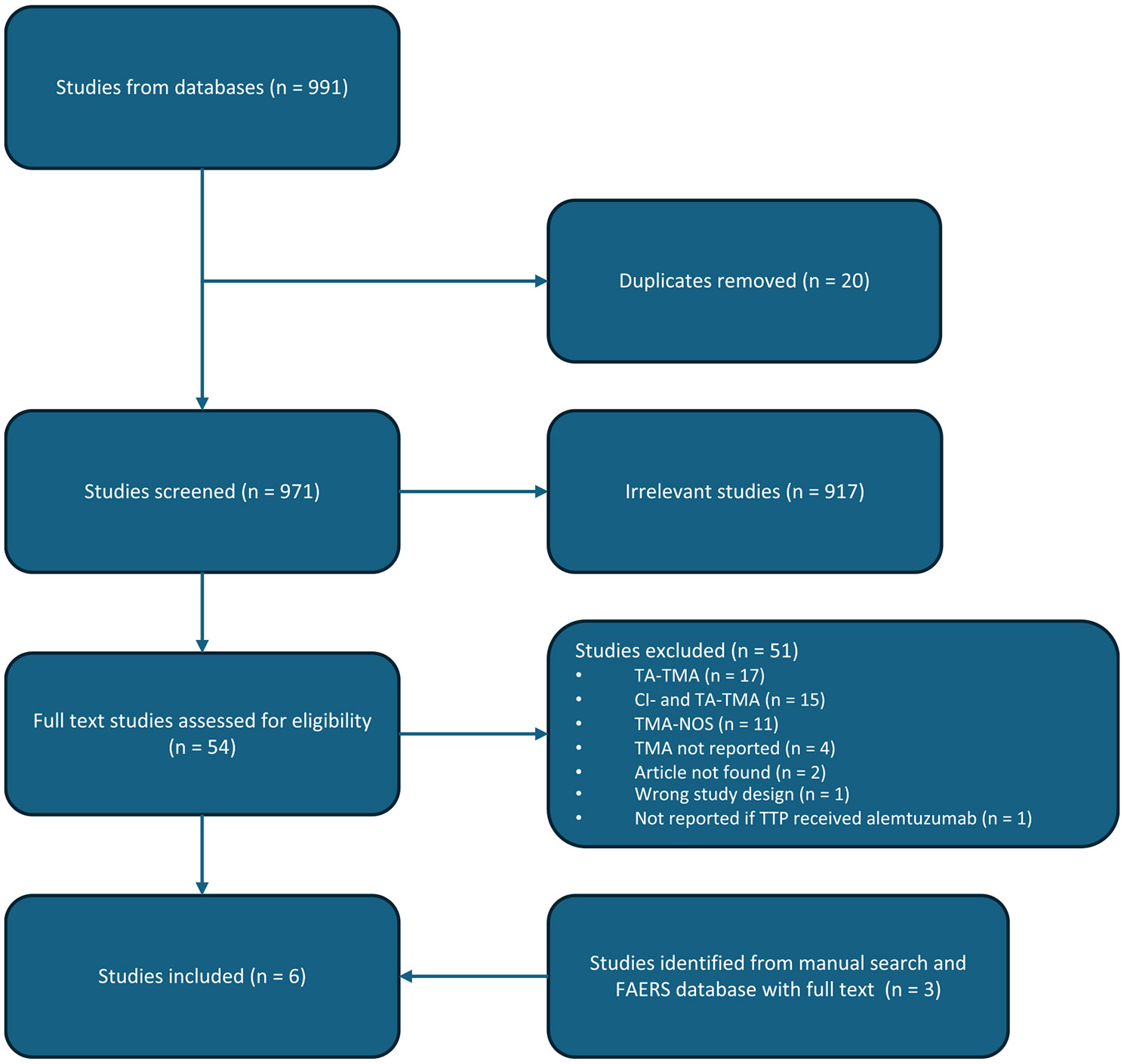
PRISMA flow diagram for systematic review. Abbreviations: TA, transplant-associated; CI, calcineurin inhibitor; TMA, thrombotic microangiopathy; NOS, not otherwise specified; TTP, thrombotic thrombocytopenic purpura.

We also systematically reviewed EMBASE and PubMed from 01 January 2000 to 03 September 2024 using the terms “alemtuzumab”, “Campath”, “Lemtrada”, “Mabcampath” “thrombotic microangiopathy”, “TMA”, “thrombotic thrombocytopenic purpura”, and “TTP”. Non-human and non-primary studies were excluded. All identified citations were uploaded to Covidence and screened against the inclusion criteria for further analysis independently by two authors (JWJ, TCB) (Fig. 1). Articles were included if they referenced at least one patient with TTP (either reported as such or defined as a TMA with an ADAMTS13 activity < 10 %) and previous or concomitant treatment with alemtuzumab. We excluded articles describing TMA in the setting of transplantation or CI use that did not contain ADAMTS13 activity or an explicit diagnosis of TTP (ie, where the authors of the original report specifically stated that TTP was diagnosed). We chose to make these systematic literature review criteria more stringent based upon our preliminary assessment of FAERS data that were limited by confounding scenarios of transplantation or CI use. Data abstracted from published cases included patient demographics, therapy indications, time from alemtuzumab administration to TTP diagnosis, associated medications, laboratory findings, and outcomes.

## 3. Results

### 3.1. FAERS database analysis

Amongst FAERS reports, Alemtuzumab represented 0.06 % (16,919/29,661,136) of all adverse events, 0.09 % (14,493/16,394,188) of all serious events, and 0.09 % (2501/2,683,911) of all deaths. The largest number of alemtuzumab-related reports were in the “General Disorders and Administration Site Conditions” (n = 7177) and “Infections and Infestations” (n = 6893) Reaction Groups, which are based on a classification of the side effect using the Medical Dictionary for Regulatory Activities (MedDRA) dictionary of reaction terms. We identified a total of 49 reports with TTP as a diagnosis, representing 0.29 % (49/16,919) of all alemtuzumab-related reports and 1.26 % (49/3899) of all alemtuzumab adverse events in the “Blood and Lymphatic System Disorders” Reaction Group. Notably, 106 reports of alemtuzumab using the FAERS code “Thrombotic Microangiopathy” were identified; 2 of these reports also included the FAERS code “TTP” and were included in the TTP analysis. The 104 remaining reports were distinct from the reports of TTP and were not included in the analysis as TMA does not necessarily imply severe ADAMTS13 deficiency. Of note, ADAMTS13 activity is not a parameter that is captured in the FAERS database.

All reports of TTP were considered serious. The mortality for all TTP reports was 18.4 % (9/49), and fatal TTP cases comprised 0.4 % (9/2501) of all alemtuzumab-associated deaths. Eight reports had corresponding references (n = 5 unique references), 80 % (4/5) of which were available for review (Table 1). Among the 5 references, 1 did not have full text available for review, 3 described patients undergoing transplantation and/or receiving CIs, and 1 described a patient with multiple sclerosis.

**Fig. 2.**
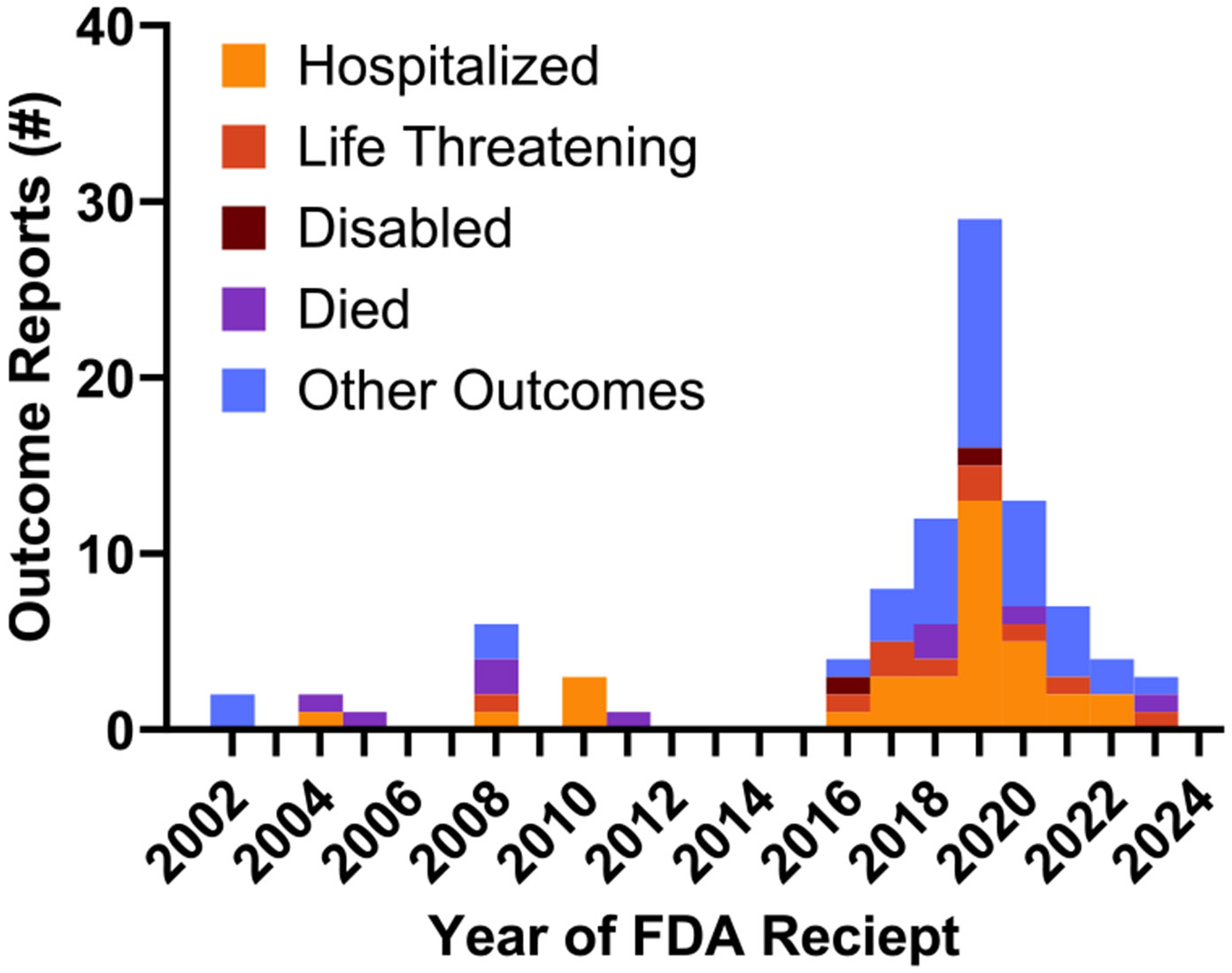
The number and outcome of reports of TTP associated with alemtuzumab use received by the US FDA by year of initial case report receipt. Note that more than one outcome may have occurred for a single report (eg, hospitalized and died).

**Fig. 3.**
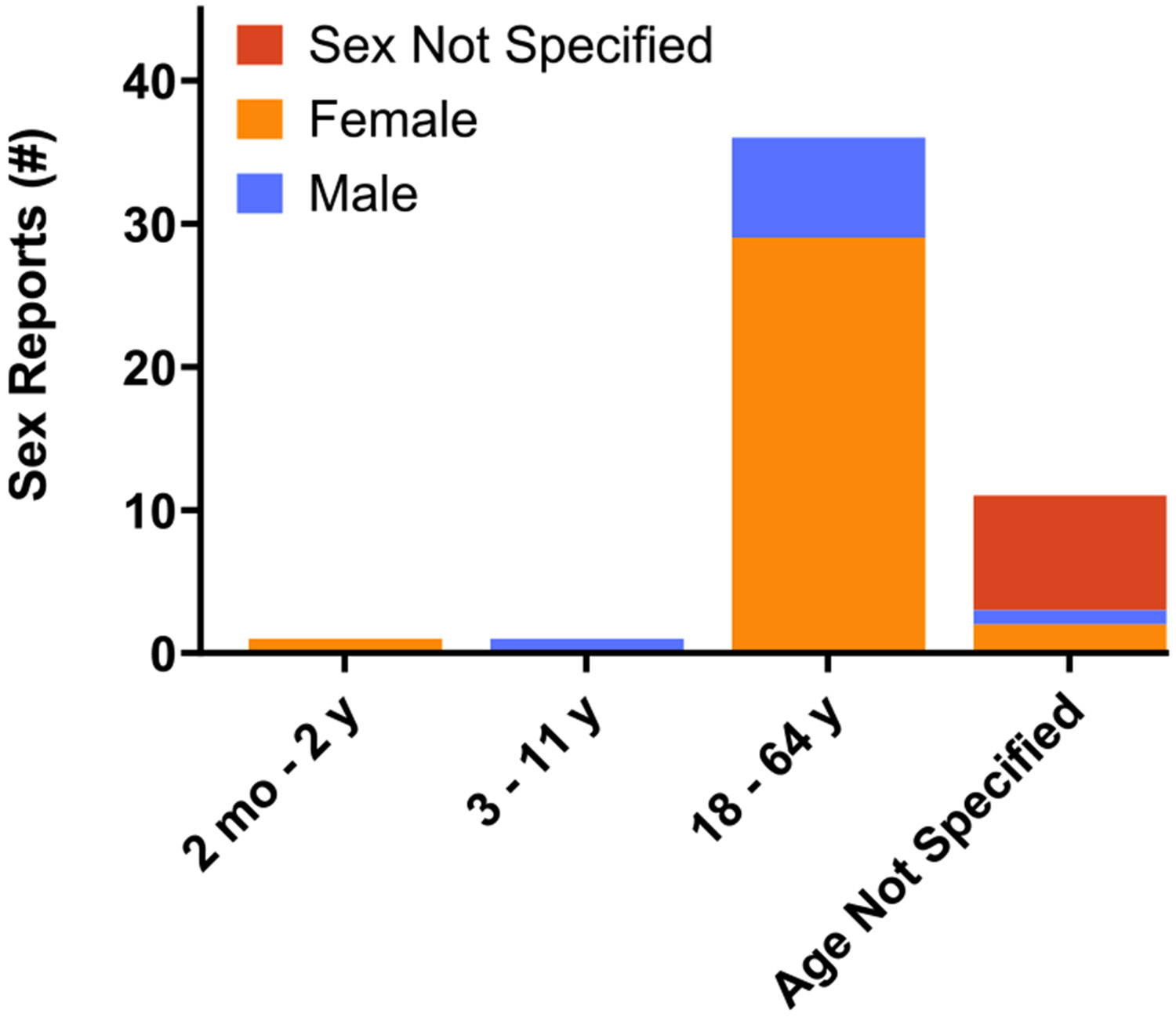
Number of reports of TTP associated with alemtuzumab received by the US FDA by sex and age group.

The number of annual reports of alemtuzumab-associated TTP received by the FDA peaked between 2018 and 2020 (Fig. 2). Most cases have been reported by healthcare professionals (n = 36), while 12 reports were made by consumers, and in one case, the identity of the reporting entity was not specified. Patient age was reported for 38 of the 49 cases, with a median age of 46 years (IQR 33–48 years, range 0–60 years). Sex was reported in 41 cases (n = 32 females, 65.3 % and n = 9 males, 18.4 %), and was not specified in 8 cases (16.3 %). Females between 18 and 64 years of age accounted for 59.2 % (29/49) of all cases (Fig. 3).

The most frequently reported indication for alemtuzumab was MS (63.3 %, 31/49), while 16.3 % (8/49) of reports were in patients for stem cell/bone marrow transplantation and graft-versus-host disease prophylaxis as an indication. In 8.2 % (4/49) of reports, the indication was “immunosuppression” and in the remaining 12.2 % (6/49) of reports, the indication was either not specified or simply stated a hematopoietic neoplasm (e.g., lymphoma, acute myeloid leukemia).

The FAERS database distinguishes between “suspected agents” and “concomitant agents”, with the former representing the medication(s) favored to most likely be potentially associated with the adverse event, while the latter category is inclusive of all medications being taken by the patient. Alemtuzumab was the sole agent of suspect in 53.1 % (26/49) of reports, while 46.9 % (23/49) of reports documented suspicion of at least one other medication in addition to alemtuzumab. In eight of these latter cases, the additional drugs of suspect reported were limited to acetaminophen and omeprazole. Concomitant use of or suspicion of at least one additional medication was documented in 77.6 % (38/49) of reports. In many of these cases, at least one chemotherapeutic and/or immunosuppressive was reported as a concomitant medication or suspect product in a variety of combinations (fludarabine, n = 8; rituximab, n = 5; mycophenolate mofetil, n = 4; cyclophosphamide, n = 4; melphalan, n = 4; methotrexate, n = 3; belatacept, n = 2). Ten reports documented concomitant or suspected use of CIs or mammalian target of rapamycin (mTOR) inhibitors, with four reports indicating 2 different CIs/mTOR inhibitors (tacrolimus, n = 7; cyclosporine, n = 6; sirolimus, n = 1; everolimus, n = 1). Cases with no suspected or concomitant products reported other than alemtuzumab represented 22.4 % (11/49) of cases.

We also included a subset analysis of only those reports for which alemtuzumab was not associated with transplantation or CI use. There were 35 reports, 27 (77.1 %) of which were reported by healthcare professionals and 8 (22.9 %) were reported by consumers. The median age was 43 years (IQR 33–46 years, range 26–60 years). Sex was reported for 31 cases: 26 females (74.3 %) and 5 males (14.3 %); sex was not specified in 4 reports (11.4 %). Two deaths were reported (5.7 % mortality).

Specific clinical and laboratory data were not available, precluding assessment of these variables. Among available references, specific patient-level data were limited. In the most detailed report available, Stoddart et al. described a patient with multiple sclerosis who developed ITP, thyroiditis, and eventually TTP [22]. The time to TTP diagnosis was 16 months following the last alemtuzumab administration.

### 3.2. Literature review

A total of 977 citations were identified in the literature search, with 54 full texts reviewed and 3 articles included in the final analysis (Fig. 1). Forty-one of the full texts reviewed were excluded on the basis of confounding clinical scenarios of transplant association, CI use, or both, and no ADAMTS13 activity reported – suggesting the possibility of transplant-associated or drug-induced TMA distinct from alemtuzumab-induced secondary TTP. Of the articles included in the final analysis, one was also present in the FAERS database [22]. Four additional studies were included from the manual search of citations in the FAERS database. The full text of one of the references in the FAERS database could not be located. The remaining three additional studies identified from the FAERS database were included on the basis of explicit association with the MedDRA term “Thrombotic Thrombocytopenic Purpura” in FAERS, despite describing patients in the setting of transplant and/or CI use.

The 6 studies available for review described a total of 8 patients, including 2 patients that were not reported in the FAERS database (both received alemtuzumab for MS) (Table 1). Three patients were treated with alemtuzumab for multiple sclerosis, two of which reported ADAMTS13 activity levels < 10 % with inhibitors. Three additional patients received alemtuzumab as a component of the conditioning regimen for lung transplantation. These three patients had also received CI therapy. Two patients received alemtuzumab for allogeneic HSCT conditioning. Discrete data points regarding TTP onset from last exposure to alemtuzumab was available for five patients and ranged from less than 3 months to 108 months (median 16 months, interquartile range 31 months). Two patients were reported deceased.

## 4. Discussion

This study found a total of 51 reports of TTP potentially associated with alemtuzumab (Table 2); 49 reports were submitted to the FAERS database since 2002, while two additional cases were identified through systematic review of the literature. While most cases were in patients receiving alemtuzumab for MS (64.7 %, 33/51), the indication for alemtuzumab was for HSCT in eight reports, and it is noted that HSCT can be associated with TMA (ie, transplant-associated TMA). In the FAERS database, alemtuzumab was listed as the sole suspected agent in 26 of the 49 cases. In 8 additional cases, the only other suspected medications listed were acetaminophen and omeprazole, which are typically not associated with secondary TTP [23]. However, concomitant CI or mTOR inhibitor use, which have been shown to induce TMA [24], was reported in 10 cases, though the ADAMTS13 activity in these patients is generally > 25 % [25,26]. Ultimately, this compilation of cases included multiple patients without potential confounders (eg, solid organ or stem cell transplantation, other medications, etc.), illustrating that, albeit rare, TTP may potentially be associated with alemtuzumab therapy, even long after administration. In line with these findings, TTP is listed on the current package insert of Lemtrada [13]; however, the 2023 American Society of Apheresis guidelines do not currently include alemtuzumab as an agent potentially associated with DI-TMA or reference it as a possible secondary cause of TTP [5].

Stoddart et al. described one case of potential alemtuzumab-associated TTP that occurred 16 months after alemtuzumab therapy [22], and Bourdin and colleagues reported a patient who developed TTP 45 months after alemtuzumab administration [9]; neither reported use of CIs or mTOR inhibitors. As such, the significant length of time that may elapse between alemtuzumab administration and the development of immune-mediated sequelae, including TTP, may contribute to the paucity of published cases that suggest a causal link. From a physiologic perspective, studies have shown that B and T cells typically recover 6–12 months following alemtuzumab therapy [27]; thus, the prolonged time frame in which autoimmune sequelae may develop is incompletely understood. However, it is possible that TTP developed in these patients irrespective of alemtuzumab administration. Granular details corroborating the reported timeframe between alemtuzumab therapy and TTP onset are unavailable in the FAERS database and may not be reported in the medical literature.

Our findings suggest that clinical trial results and published reports may underestimate the number of potential cases. This may be a result of patients being lost to follow-up or clinical trial cessation before TTP presentation given the long latency between drug-exposure and disease onset. Long-term studies assume these limitations, and refer to this gap as missing data, an important source of bias in clinical trials [28]. Researchers performing trials for patients receiving alemtuzumab should include approaches to monitor for TTP in these subjects as recommended in the package insert (via complete blood count to assess hemoglobin and platelet levels) [13], with reflex testing as necessary (eg, peripheral blood smear, hemolysis biomarkers, ADAMTS13 activity). As it is impossible to conduct pharmaceutical trials indefinitely, reporting databases such as FAERS offer a unique and vital resource for identifying late manifestations of serious events that may be treatment-related.

In addition to our findings, the possibility of underestimation of potential alemtuzumab-related TTP cases is further supported by a review of pharmacovigilance data in which reports of “drug-induced TTP” were analyzed [29]. The authors identified seven cases of TTP possibly associated with alemtuzumab in the United Kingdom’s Medicines and Healthcare products Regulatory Agency (MHRA) database, and 13 potential cases in the European Medicines Agency’s pharmacovigilance system [29]. The combined results of all studies illustrate that alemtuzumab has been implicated as a possible factor in multiple cases of secondary TTP since its introduction.

It is important to note that there may be overlap in DI-TMA and drug-induced or secondary TTP, wherein the latter is defined by a mechanistic association (ie, a severe acquired deficiency in, or inhibition of, ADAMTS13 activity), while DI-TMA is defined by a clinical association (ie, concurrent use of a drug) with mechanistic diversity (eg, autoimmunity, drug-dependent antibodies, or endothelial damage). This distinction may complicate comparisons of secondary TTP across studies. For example, although we did not include it in our analysis, we identified one case of TMA following alemtuzumab administration in a patient with MS (in the absence of other potential causative agents) with an ADAMTS13 activity of 57 % [30]. This raises the question of whether alemtuzumab may be associated with the development of TMA via multiple mechanisms, including through the induction of autoantibodies (which may target ADAMTS13, ie, TTP), as well as independent of ADAMTS13 autoantibody formation. These different mechanisms could be influenced by a variety of factors, including genetics and environmental elements [31]. Regardless, apheresis practitioners should be aware of these possible associations when evaluating patients currently taking alemtuzumab or with a history of alemtuzumab exposure.

One limitation of the FAERS database, similar to other pharmacovigilance systems, is that it does not confirm a causal relationship [32]. As the FAERS database does not provide clinical information or laboratory results for individual patient reports, assessment of causality or the strength of the potential association between alemtuzumab and TTP could not be performed. Further, as ADAMTS13 activity is not a parameter in the FAERS database, and therefore was not reported for FAERS cases except where the corresponding literature reports were available, there is the possibility that cases referred to as “TTP” were included despite normal ADAMTS13 activity. However, we attempted to mitigate this by only including cases with the FAERS code “TTP”, whereas 104 distinct cases of “Thrombotic Microangiopathy” without a diagnosis of TTP (and theoretically therefore without a significant reduction in ADAMTS13 activity) were not included. Likewise, data validation was not performed, as we relied on the accuracy of the FAERS database maintained by the US FDA. As with many pharmacovigilance databases, the data are presumed to be valid, but cannot be definitively determined as such. Nevertheless, numerous studies have utilized the FAERS and related databases (eg, the US Vaccine Adverse Event Reporting System) to elucidate potential rare adverse events associated with drugs and vaccines [33-35], and prior studies have shown that the FAERS database has moderate sensitivity in detecting adverse events [36]. When a medical literature review is combined with analysis of the FAERS database, the ability to detect a signal improves, enhancing drug safety surveillance [37].

Another limitation includes the etiology of the disease itself. The immune-mediated form of TTP is an autoimmune condition and many of the described patients carried a primary autoimmune disease diagnosis; therefore, it is possible that the development of TTP may have been related to the underlying autoimmune condition, a combination of the autoimmune condition and alemtuzumab, or neither (ie, idiopathic). Immune thrombocytopenia has also been described in association with alemtuzumab [38]; as such, we attempted to use a strict definition of TTP in the cases where clinical and laboratory information were available [39], though these were scarce. Furthermore, reporting of cases or systematic literature reviews cannot be used to estimate incidence or prevalence rates as the number of patients treated with alemtuzumab during this time-period is unknown. Nevertheless, TTP is a rare condition, with an incidence of approximately one new case per one million persons per year [40]. Additionally, while approximately 900,000 individuals in the US had MS as of 2017 [41], alemtuzumab is typically reserved for patients with aggressive disease or as a second- or third-line therapy in patients with relapsing forms [42]. This is reflected by a study of the IBM^®^MarketScan^®^ claims database, which identified 341 patients treated with a disease modifying therapy and switched to alemtuzumab between January 1 2013 and December 2019 [43], suggesting that the number of patients with MS treated with alemtuzumab in the US annually is relatively low compared to other agents. As such, the scope of our investigation permits analysis of infrequent adverse events not detected in clinical trials or small studies.

In summary, the results of pharmacovigilance database analyses support the current package insert for alemtuzumab where TTP is listed as a “warning and precaution”, and suggest that cases of alemtuzumab-associated TTP may be more frequent than implied by the few published case reports. As such, further research into this possible association is warranted. Until more definitive data are available, healthcare professionals should be cognizant of this possible adverse event that may occur even for an extended period after cessation of alemtuzumab therapy. This is particularly important for apheresis practitioners given that the ASFA guidelines do not discuss alemtuzumab as a potential precipitant, which may be considered for inclusion in subsequent versions. Healthcare professionals may also question prior alemtuzumab use in patients with TTP, and ensure these cases are reported to the manufacturer and the FDA when discovered.

## Figures and Tables

**Table 1:** Details of published studies referenced in the FAERS database and studies identified in the literature review.

Ref.	Number of patients	Indication for alemtuzumab	Latency to TTP diagnosis from alemtuzumab administration	Platelet count nadir	ADAMTS13 activity and autoantibody quantitation	TTP treatment	Time to response	Achievement of remission
Iasella CJ, Winstead RJ, Moore CA, Johnson BA, Feinberg AT, Morrell MR Et al. Maintenance Belatacept-Based Immunosuppression in Lung Transplantation Recipients Who Failed Calcineurin Inhibitors. Transplantation. 2018;102(1):171–177	3	Lung transplantation	Patient 1: 3 months post-alemtuzumab induction Patient 2: 14 months post-alemtuzumab induction Patient 3: 108 months post-alemtuzumab induction	Patient 1: NR Patient 2: NR Patient 3: < 20 × 10^9^/ L	Patient 1: NR Patient 2: NR Patient 3: NR	Patient 1: TPE and rituximab Patient 2: NR Patient 3: TPE and IVIG	Patient 1: NR Patient 2: NR Patient 3: NR	Patient 1: Yes Patient 2: Yes Patient 3: Yes
Stoddart L, Shah N, Hassan S, Andrze-Jewski C. Thrombotic thrombocytopenic purpura (TTP) in a patient with multiple sclerosis receiving alemtuzumab. Transfusion. 2018;58 (Supplement2):95A–96A	1	Multiple sclerosis	16 months after last alemtuzumab dose	6 × 10^9^/L	ADAMTS13 activity < 5 % ADAMTS13 autoantibody 1.2 BU	TPE (21 procedures) and rituximab (4 doses)	NR	Yes
Young K L, Howard D, Lekakis L. Use of alemtuzumab as graft-versus-host disease prophylaxis in myeloablative unrelated donor transplants Journal of Clinical Oncology. 2008;26 (SUPPL ABSTR 7045).	1	Graft-versus-host disease prevention with myeloablative conditioning for unrelated stem cell transplant	< 100 days after alemtuzumab administration	NR	NR	NR	N/A	No (death)
Rauma I, Mustonen T, Seppä JM, et al. Safety of alemtuzumab in a nationwide cohort of Finnish multiple sclerosis patients. *J Neurol.* 2022;269 (2):824–835. *Not reported in FAERS database	1	Multiple sclerosis	NR	NR	NR	Intensive care and TPE	NR	NR
Bourdin V, Fossé Q, Lambotte O, et al. Alemtuzumab-induced immune-mediated thrombotic thrombocytopenic purpura: A newly described drug-related autoimmune disease. *Br J Haematol.* 2024;204(4):1459–1463. *Not reported in FAERS database	1	Multiple sclerosis	45 months following alemtuzumab	5 × 10^9^/L	< 5 % (FRETS-VWF73 assay, Peptide Institute Inc., Osaka, Japan) Anti-ADAMTS13 IgG, titer > 90 U/mL (Technozym^®^ ADAMTS13 INH ELISA, Technoclone, Vienna, Austria)	TPE, corticosteroids, rituximab, and caplacizumab	3 months (clinical remission)	Yes
Ingram W, Devereux S, Das-Gupta EP, et al. Outcome of BEAM-autologous and BEAM-alemtuzumab allogeneic transplantation in relapsed advanced stage follicular lymphoma. *Br J Haematol.* 2008;141(2):235–243.	1	Part of conditioning regimen: BEAM (BCNU [carmustine], cytarabine, etoposide, melphalan)-alemtuzumab allogeneic HSCT (BEAM-allo)) for relapsed advanced stage follicular lymphoma	NR	NR	NR	NR	N/A	No (death)
Mariann R, Barta A, Goda V, et al. Acute graft versus host (AGVHD) disease treatment with extracorporalis photopheresis, 2004–2011 Febr. Haematology-Transfusiology. 2011;44 (1):61–62.	Text not available for review							

**Abbreviations**: ADAMTS13, a disintegrin-like metalloproteinase with thrombospondin motif type 1 member 13; TPE, therapeutic plasma exchange; IVIG, intravenous immune globulin; NR, not reported; N/A, not applicable; BU, Bethesda units; TTP, thrombotic thrombocytopenic purpura; FRETS-VWF73, fluorescent resonance energy transfer-von Willebrand factor 73; ELISA, enzyme linked immunosorbent assay; FAERS, United States Food & Drug Administration Adverse Event Reporting System.

**Table 2:** Details of all reports identified in the FAERS database and literature review combined (n = 51).

Variable	Median (IQR) or no. (%)
Age (n = 39)	46 (34–48)
Sex	
Female	32 (62.7 %)
Male	10 (19.6 %)
Unknown/not reported	9 (17.6 %)
Alemtuzumab indication	
Multiple sclerosis	33 (64.7 %)
Stem cell/bone marrow transplantation and GVHD prophylaxis	8 (15.7 %)
Other	10 (19.6 %)
Outcome	
Alive	41 (80.4 %)
Deceased	9 (17.6 %)
Unknown/not reported	1 (2.0 %)

Abbreviations: GVHD, graft-versus-host disease; FAERS, United States Food & Drug Administration Adverse Event Reporting System.
